# p38/TF/HIF-*α* Signaling Pathway Participates in the Progression of CIPN in Mice

**DOI:** 10.1155/2019/5347804

**Published:** 2019-07-10

**Authors:** Yang Yang, Liang Hu, Chaoyu Wang, Xing Yang, Ling Song, Chunyi Jiang, Yan Li, Tianxi Li, Wen-Tao Liu, Jifeng Feng

**Affiliations:** ^1^The Affiliated Cancer Hospital of Nanjing Medical University & Jiangsu Cancer Hospital & Jiangsu Institute of Cancer Research, Nanjing, Jiangsu 210009, China; ^2^Jiangsu Key Laboratory of Neurodegeneration, Department of Pharmacology, Nanjing Medical University, Nanjing 210029, China; ^3^Department of Oncology, Shandong Qianfoshan Hospital, Jinan 250014, Shandong Province, China

## Abstract

Chemotherapy induced peripheral neuropathy (CIPN) is a serious adverse effect of chemotherapeutics with limited pathogenetic mechanism been known. Whether microcirculatory disturbance is involved in CIPN has not been reported. Considering that tissue factor (TF) is an endogenous coagulation factor, we hypothesize CIPN may be induced by the high expression of TF in macrophages and sciatic nerve, which induces the molecular signal related to ischemia and hypoxia. Oxaliplatin (L-OHP) was used to establish CIPN model. Von Frey Hairs was used to measure nociception. The murine macrophage cell line Raw 264.7 was used for cell experiments. Gelatin zymography and western blotting were used to measure the activity or expression of protein. TF expression and MMP-9/2 activity in sciatic nerve and blood are significantly increased by L-OHP. L-OHP increased the release of HSP70 from macrophage and enhanced the expression of p-p38 and HIF-1*α* in vivo and in vitro. Hirudin significantly suppressed the overexpression of p38, HIF-1*α* and activation of MMP-9/2 induced by L-OHP and attenuated CIPN in mice. This study suggests that a novel HSP70-TLR-4-p38-TF-HIF-1a axis may play a pivotal role in the pathological process of CIPN. It is also shown that the use of anticoagulant Hirudin can inhibit the above mechanisms and improve CIPN.

## 1. Introduction

Chemotherapy is widely applied for malignant tumors treatment. However, Chemotherapy-induced peripheral neurotoxicity (CIPN) is one of the most severe side effects of Chemotherapy, which is a progressive and often irreversible condition featuring pain, numbness, tingling and sensitivity to cold in the hands and feet. About 60% of CIPN patients suffering develop insomnia, anxiety and depression symptoms, which seriously affects the patient's mental state and ultimately hinders the treatment of cancer [1]. As a major dose-limiting side effect, many chemotherapeutic agents could cause CIPN [2], such as platinum (oxaliplatin), paclitaxel (paclitaxel), vinblastine (Changchunxin Alkali) and other first-line drugs during the course of chemotherapy [3, 4].

Despite decades of extensive studies on mechanisms underlying CIPN, knowledge of its pathogenetic aspects is still very limited [5]. Studies suggested that neuronal mechanisms, such as nerve damage [6], ion channel changes, demyelination [7], high concentration of intracellular reactive oxygen species [8], mitochondrial dysfunction [9] and DNA damage [10], play pivotal roles in the initiation of CIPN. However, compelling evidences recently show that neuroinflammation mechanisms, especially macrophage, mediate inflammation and also participate in the development and maintenance of CIPN [10]. The expression of monocyte chemoattractant protein-1 (MCP-1) and its receptor CCR2 in DRG increased significantly during PTX-induced mechanical allodynia, accompanied by increased infiltrated macrophage and up-regulated tumor necrosis factor-alpha and interlukin1-*β* expression in DRG [11].

In addition to releasing inflammatory factors, chemotherapy also can induce macrophages to express Tissue factor (TF) and participate in the process of thrombosis [12]. TF is the main mechanism of exogenous coagulation [13], binding to Factor VII and rapidly initiating the coagulation leading to formate thrombus and microthrombus [14, 15]. Previous studies showed that chemotherapy drug significantly increases the expression of TF and decreases the expression of TF pathway inhibitor (TFPI) in macrophages, platelets and vascular endothelium [16, 17]. As a highly energy consuming tissue, the nervous system is very sensitive to the change of oxygen supply. High expression of TF may induce microthrombosis around peripheral nerves, especially in the microenvironment surrounding the sciatic nerve, triggering hypoxia-related molecular events such as elevation of hypoxia-inducible factors [18]. Hypoxia-inducible factors (HIFs) are transcription factors that respond to decreases in available oxygen in the cellular environment, or hypoxia. Activation of HIF-1*α* may enhance the expression of many downstream cytokines [19], such as vascular endothelial growth factor (VEGF), and matrix metalloproteinases (MMPs), which have been extensively demonstrated to be involved in peripheral sensitization of chronic pain [20, 21].

Therefore, we hypothesize that chemotherapy-induced TF may be involved in CIPN by increasing HIF-1*α* and MMPs activation through thrombosis-mediated circulatory disturbance in the sciatic nerve microenvironment [12]. Based on the above hypothesis, this study will investigate the changes of TF-HIF-1*α*-MMPs axis in macrophage and sciatic nerve during L-OHP-induced CIPN. At the same time, we will further explore the upstream signal of TF induced by chemotherapy, that is, whether the release of HSP70, another early warning molecule (DAMPs), will be increased by chemotherapeutic drugs, and whether it can mediate the increase of TF through TLR-4 signal. Similar to the release of HSP70, L-OHP also significantly increased the phosphorylation of p38 in vivo and in vitro. Moreover, we will further investigate the possibility of using an anticoagulant hirudin to disrupt the TF-HIF-1*α*-MMPs axis and alleviate CIPN in mice.

## 2. Materials and Methods

### 2.1. Ethics Statement

All the experiments were strictly performed in accordance with the regulations of the ethics committee of the International Association for the Study of Pain and the Guide for the Care and Use of Laboratory Animals (The Ministry of Science and Technology of China, 2006). All animal experiments were approved by Nanjing Medical University Animal Care and Use Committee and were designed to minimize suffering and the number of animals used.

### 2.2. Animals and Chemotherapy-Induced Peripheral Neuropathy Model

Adult male C57BL/6 mice (20-25 g wt) were provided by the Model Animal Research Center of Nanjing University, Nanjing, China. Animals are preadapted to the environment with five to six per cage under pathogen-free conditions with soft bedding under controlled temperature (22 ± 2°C) and photoperiods (12:12-h light–dark cycle) for at least 2 days. Animals were randomly divided into groups. Oxaliplatin (L-OHP, 4 mg/kg) was administrated by intraperitoneal injection on 1st, 4th, 8th and 11th day to make mice CIPN model [22]. Hirudin (10 mg/kg,* i.p.*) was administrated once per day till 14 days [23]. The first administration of hirudin was from the day before the CIPN model was built.

### 2.3. Drugs and Reagents

Antibody for *β*-actin was purchased from Sigma (St. Louis, MO). Antibodies for hypoxia inducible factor-1*α* (HIF-1*α*), heatshockprotein70 (HSP70), phosphorylated p38 (Tyr182) were purchased from Cell Signaling Technology (Beverly, MA). TF was purchased from Santa Cruz Biotechnology (Dallas, Texas, USA.). Secondary antibodies were purchased from Sigma (St. Louis, MO). L-OHP was purchased from Jiangsu heng rui medicine (Lianyungang, China). Gelatin was purchased from Amresco (Solon, OH, USA). Zymogram renaturing buffer and Zymogram developing buffer were purchased from Novex (Carlsbad, CA, USA). All other reagents were purchased from Sigma-Aldrich (St. Louis, MO, USA). Fetal bovine serum (FBS) was purchased from Gibco, and other cell culture media and supplements were purchased from HyClone (Logan, UT, USA). Coomassie brilliant blue G250 was purchased from Beyotime (Shanghai, China). Dimethyl sulfoxide (DMSO) was purchased from Sigma Chemical Co (St. Louis, MO). All other chemicals were purchased from Sigma Chemical Co (St. Louis, MO). Hirudin was purchased from Genscript (Nanjing, China). p38 inhibitor SB 203580 was purchased from MedChemExpress (NJ, USA).

### 2.4. Gelatin Zymography

Animals were anesthetized deeply with 10% chloral hydrate (10 mg/kg, i.p.). Sciatic nerves were rapidly dissected and homogenized in 1% NP40 lysis. 300-500 *μ*g of Sciatic nerve protein and blood plasma per lane was loaded into the wells of precast gels (8% polyacrylamide gels containing 0.1% gelatin). After electrophoresis, each gel was incubated with 50 ml of developing buffer for 48 hours (37.5 centigrade degree) in shaking bath. Then the gels were stained with coomassie brilliant blue (1%, with 10% acetic acid, 10% isopropyl alcohol, diluted with dd H_2_O).

### 2.5. Western Blotting

Samples (sciatic nerve, blood and RAW264.7) were collected and washed with ice-cold PBS before being lysed in radio immunoprecipitation assay (RIPA) lysis buffer [Beyotime, Shanghai, China; 50 mmol/L Tris (pH 7.4), 150 mmol/L NaCl, 1% Triton X-100, 1% sodium deoxycholate, 0.1% sodium dodecyl sulfate, 1 mmol/L phenylmethylsulfonyl fluoride, 0.15 U/mL aprotinin, and 1 mg/mL pepstatin] and then sample lysates were separated by SDS-PAGE and electrophoretically transferred onto polyvinylidene fluoride membranes (Millipore Corp, Bedford, MA, USA). The membranes were blocked with 10% whole milk in TBST (Tris–HCl, NaCl, Tween 20) for 2 h at room temperature, probed with primary antibody at 4°C overnight. The primary antibodies used are HSP-70 (1:1000), TF (1:1000), HIF-1*α* (1:1000), p-p38 (1:1000), *β*-actin (1:5000). Then they were incubated with horseradish peroxidase-coupled secondary antibodies from Sigma (St. Louis, MO). Data were acquired with the Molecular Imager (Gel DocTM XR, 170–8170) and analyzed with Quantity One-4.6.5 (BioRad Laboratories, Berkeley, CA, USA).

### 2.6. Behavioral Analysis

Mechanical sensitivity was measured by Von Frey Hairs (Woodland Hills, Los Angeles) test. Animals were placed in boxes set on an elevated metal mesh floor and were allowed 30 min for habituation before testing. The plantar surface of each hind paw was stimulated with a series of von Frey hairs with logarithmically incrementing stiffness perpendicularly to the plantar surface. Each mouse was tested for three times and the average of the threshold was measured. Behavioral tests were performed blindly.

### 2.7. Cell Preparation and Stimulation

RAW264.7 cells were maintained in humidified 5% CO_2_ at 37°C in Dulbecco's modified Eagle's Medium (DMEM) supplemented with 10% (v/v) FBS, penicillin (100 U/ml), and streptomycin (100 U/ml). For inducing inflammasome activation, 10^5^ cells were plated in 6-well plate overnight and then cells were treated with L-OHP (1 *μ*M) for 2, 4, 8 h. Cell extracts and precipitated supernatants were analyzed by immunoblotting.

### 2.8. Immunofluorescence

RAW264.7 cells were plated in class bottom cell culture dishes and treated with L-OHP (1 *μ*M) for 4 h with or without p38 inhibitor SB 203580(10 *μ*M). Then RAW264.7 cells were fixed with ice-cold methanol and were permeabilized with 0.25% Triton X-100/PBST. After blocking with 1% bovine serum albumin (BSA) in PBST for 1 h, the coverslips with RAW264.7 cells were incubated for 2 h at room temperature with the TF antibody diluted in 1% BSA (1:50). Then the coverslips were exposed to the fluorescein isothiocyanate (FITC)-conjugated anti-rabbit IgG (1:100, at room temperature for 1 h) and then were rinsed three times with PBS. Finally, the coverslips were stained with 1 *μ*g/mL DAPI (4′,6-diamidino-2-phenylindole, a fluorescent DNA dye to mark nucleus) for 1 min. Confocal microscopy analysis was carried out using Olympus FV1000 confocal system.

### 2.9. Statistical Analyses

SPSS Rel 15 (SPSS Inc., Chicago, IL, USA) was used to conduct all the statistical analyses. Data were statistically evaluated by two-way analysis of variance (ANOVA) followed by Bonferroni post hoc tests. The mean fluorescent pixels of TF were measured by Image Pro Plus 6.0 (Media Cybernetics, Silver Spring, MD, USA). Results were represented as mean ± standard error of the three independent experiments. Results described as significant were based on a criterion of p < 0.05.

## 3. Results

### 3.1. L-OHP Induced Mechanical Allodynia in Mice and Enhanced Over-Expression of TF in Sciatic Nerve and Macrophages

**Figure 1:**
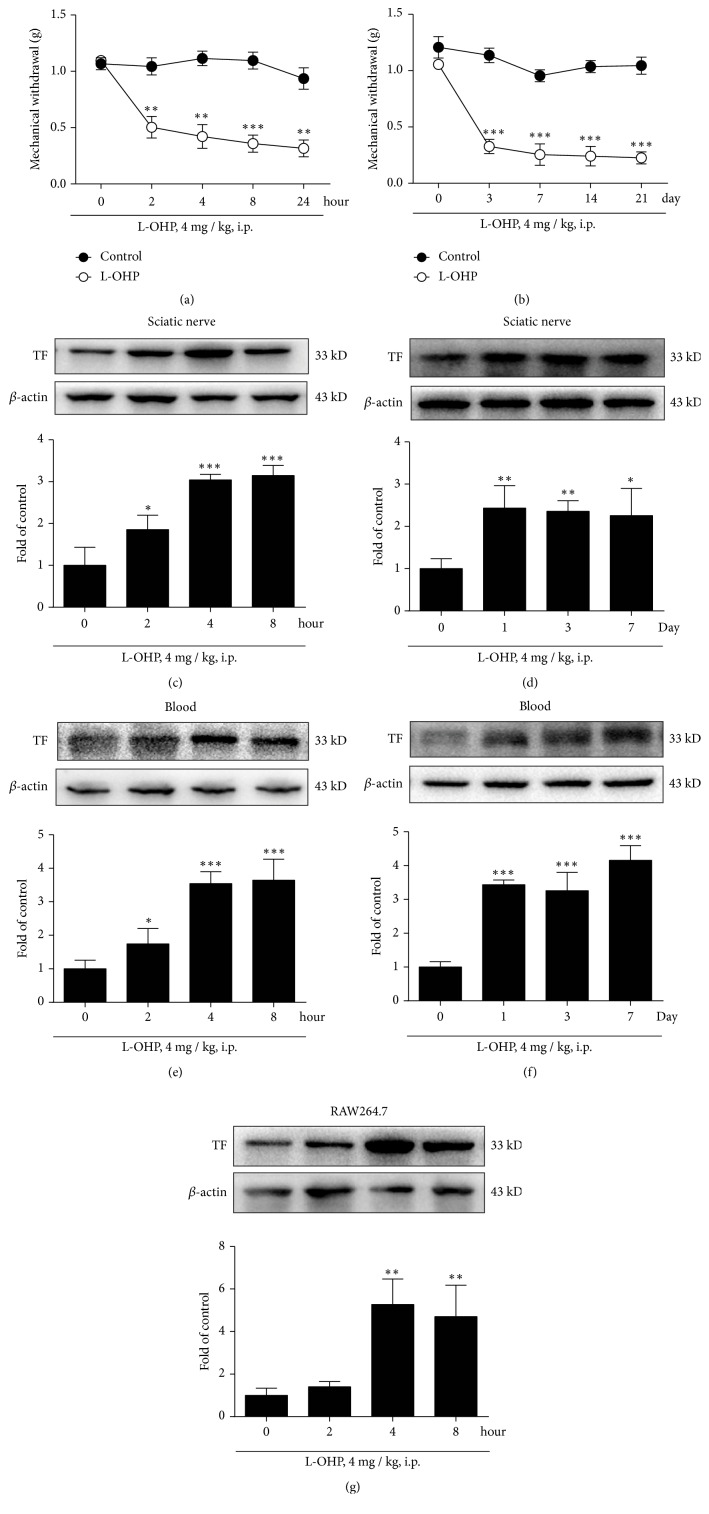
L-OHP induced mechanical allodynia in mice and enhanced over-expression of TF in sciatic nerve and macrophages. (a) Mechanical thresholds after a single-injection of vehicle or L-OHP (4 mg/kg,* i.p.*) (n = 6). (b) Mechanical thresholds after four times injection of vehicle or L-OHP (4 mg/kg,* i.p.*, injected on 1, 4, 8 and 11 day) (n = 6). (c) TF expression in the sciatic nerve after a single injection of vehicle or L-OHP (4 mg/kg,* i.p.*) at 4 and 8 h (n = 4). (d) TF expression in the sciatic nerve after two times injection of vehicle or L-OHP (4 mg/kg,* i.p.*, injected on 1 and 4 day) (n = 4). (e) TF expression in the blood after a single injection of vehicle or L-OHP (4 mg/kg,* i.p.*) at 4 and 8 h (n = 4). (f) TF expression in the blood after two times injection of vehicle or L-OHP (4 mg/kg,* i.p.*, injected on 1 and 4 day) (n = 4). (g) TF expression in RAW264.7 cells after vehicle or L-OHP (10 *μ*M) administration (n = 4). Significant difference was revealed following one-way or two-way ANOVA (^∗^P < 0.05, ^∗∗^P < 0.01, ^∗∗∗^P < 0.001* vs.* control; Bonferroni post hoc tests).

L-OHP (4 mg/kg,* i.p.*) was used to establish mice CIPN model. Firstly, we measured the mechanical thresholds of L-OHP treated mice using the Von Frey test. As shown in Figures 1(a) and 1(b), L-OHP decreased the mechanical thresholds of mice from 1.095 ± 0.036 g to 0.315 ± 0.075 g at 24h after a single-injection (Figure 1(a)) and from 1.054 ± 0.043 g to 0.225 ± 0.053 g on 21 days after four times injection (Figure 1(b)). Secondly, we measured the expression of TF, which is the major player in initiation of the blood coagulation process. As shown in Figures 1(c)–1(g), L-OHP (4 mg/kg,* i.p.*) significantly increased the expression of TF at 2, 4 and 8h after a single-injection (Figures 1(c) and 1(e)) or on 1, 3 and 7 d after two times injection (Figures 1(d) and 1(f)) in sciatic nerve and blood. In order to evaluate the direct effect of L-OHP on the expression of TF on macrophages, we treated RAW264.7 with L-OHP with different dosage then detected the expression of TF on macrophages by western blotting. The results showed that L-OHP could significantly increase the expression of TF on macrophages (Figure 1(g)).

### 3.2. L-OHP Treatment Promoted Macrophage HSP70 Release and p38 Activation In Vivo and In Vitro

**Figure 2:**
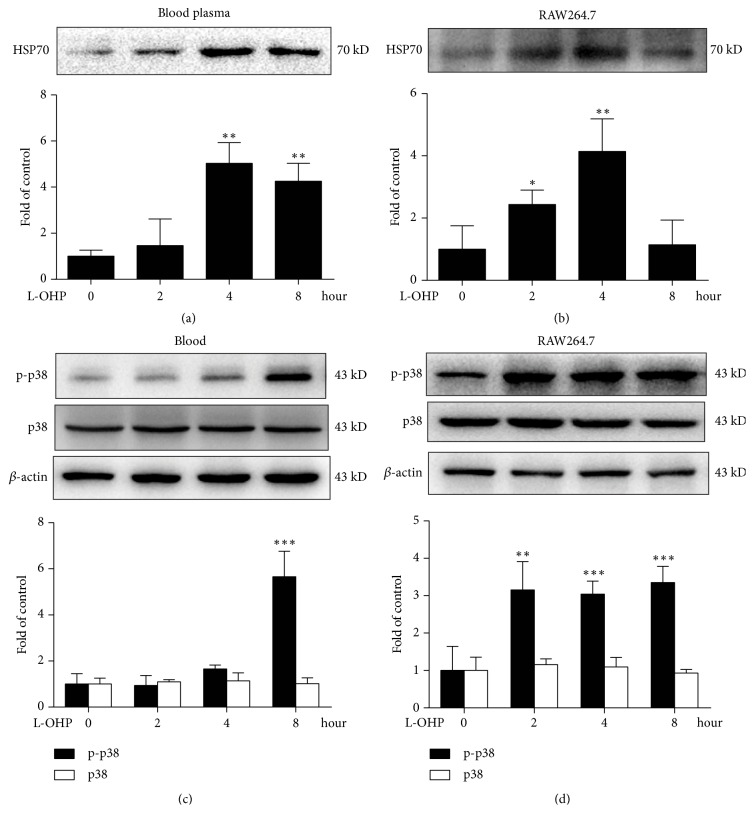
L-OHP treatment promoted macrophage HSP70 release and p38 activation in vivo and in vitro. (a) HSP70 expression in blood plasma after a single injection of vehicle or L-OHP (4 mg/kg,* i.p.*) at 2, 4 and 8 h (n = 4). (b) HSP70 expression in the culture medium of RAW264.7 cells after vehicle or L-OHP 10 *μ*M) administration (n = 4). (c) p-p38 and p38 expression in the blood plasma after a single injection of vehicle or L-OHP (4 mg/kg,* i.p.*) at 2, 4 and 8 h (n = 4). (d) p-p38 and p38 expression in the culture medium of RAW264.7 cells after vehicle or L-OHP (10 *μ*M) administration (n = 4). Significant difference was revealed following one-way ANOVA (∗*P* < 0.05, ∗∗*P *< 0.01, ∗∗∗*P *< 0.001* vs*. control; Bonferroni post hoc tests).

Previous studies have shown that activation of p38 induced by TLR4 is an important upstream signal of TF expression in macrophages. TLR-4 ligands are classified into endogenous and exogenous ligands. LPS is a classic exogenous ligand of TLR-4 [24]. Our results showed that the expression of TF on macrophages increased rapidly after four hours of L-OHP treatment. Chemotherapy-induced immunosuppression and intestinal barrier destruction-induced LPS invasion seem to be difficult to fully explain such rapid TLR-4 signal activation, so we hypothesize whether chemotherapy induces an increase in serum levels of endogenous TLR-4 agonists. HSP70 is an important endogenous agonist of TLR-4, and it can be rapidly released into the extracellular environment under cellular stress. Next we examined the expression level of HSP70 in serum after L-OHP treatment (Figures 2(a) and 2(b)). It shows that L-OHP can rapidly promote the expression level of HSP70 in blood. At the same time, we used RAW264.7 to verify the results of in vivo experiments. The results showed that L-OHP could induce HSP70 release within two hours in vitro, earlier than the onset time of TF expression. We also examined p38, a downstream signal of TLR-4 and an upstream signal of TF. Similar to the release of HSP70, L-OHP also significantly increased the phosphorylation of p38 in vivo and in vitro, but did not affect the expression of p38 (Figures 2(c) and 2(d)).

### 3.3. L-OHP Induce the Over Expression of HIF-1*α* and Activation of MMP-9/2 in Sciatic Nerve

**Figure 3:**
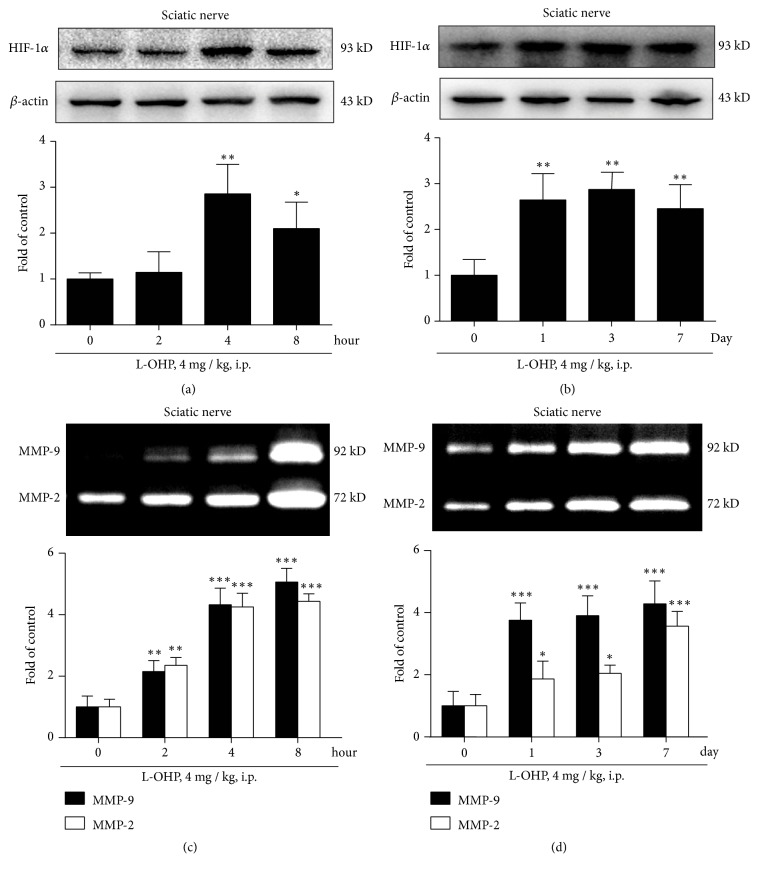
L-OHP induce the expression of HIF-1*α* and activation of MMP-9/2 in sciatic nerve. (a) HIF-1*α* expression in sciatic nerve after a single injection of vehicle or L-OHP (4 mg/kg,* i.p.*) at 2, 4 and 8 h (n = 4). (b) HIF-1*α* expression in the sciatic nerve after two times injection of vehicle or L-OHP (4 mg/kg,* i.p.*, injected on 1 and 4 day) (n = 4). (c) MMP-9/2 activity in sciatic nerve after a single injection of vehicle or L-OHP (4 mg/kg,* i.p.*) at 2, 4 and 8 h (n = 4). (d) MMP-9/2 activity in the sciatic nerve after two times injection of vehicle or L-OHP (4 mg/kg,* i.p.*, injected on 1 and 4 day) (n = 4). Significant difference was revealed following one-way ANOVA (∗*P* < 0.05, ∗∗*P *< 0.01, ∗∗∗*P *< 0.001* vs*. control; Bonferroni post hoc tests).

After confirming that L-OHP could induce TF overexpression and p38 activation in macrophages, we then examined the expression of HIF-1*α* and activity of MMP-9/2 in the sciatic nerve. As shown in Figure 3, the expression of HIF-1*α* in sciatic nerve was significantly increased after single administration of L-OHP in a time-dependent manner. It can increase 3-fold in four hours (Figure 3(a)). After repeated L-OHP treatment, the high level of HIF-1*α* expression lasted for more than a week (Figure 3(b)). At the same time, we also detected the activity of MMP-9/2 in the sciatic nerve (Figure 3(c)). The results were consistent with the expression trend of HIF-1*α*. L-OHP could induce a rapid and significant increase of MMP-9/2 in the sciatic nerve within four hours (Figure 3(d)).

### 3.4. Hirudin Can Significantly Suppress the Overexpression of p38, HIF-1*α* and MMP-9/2 Induced by L-OHP and Attenuate CIPN

**Figure 4:**
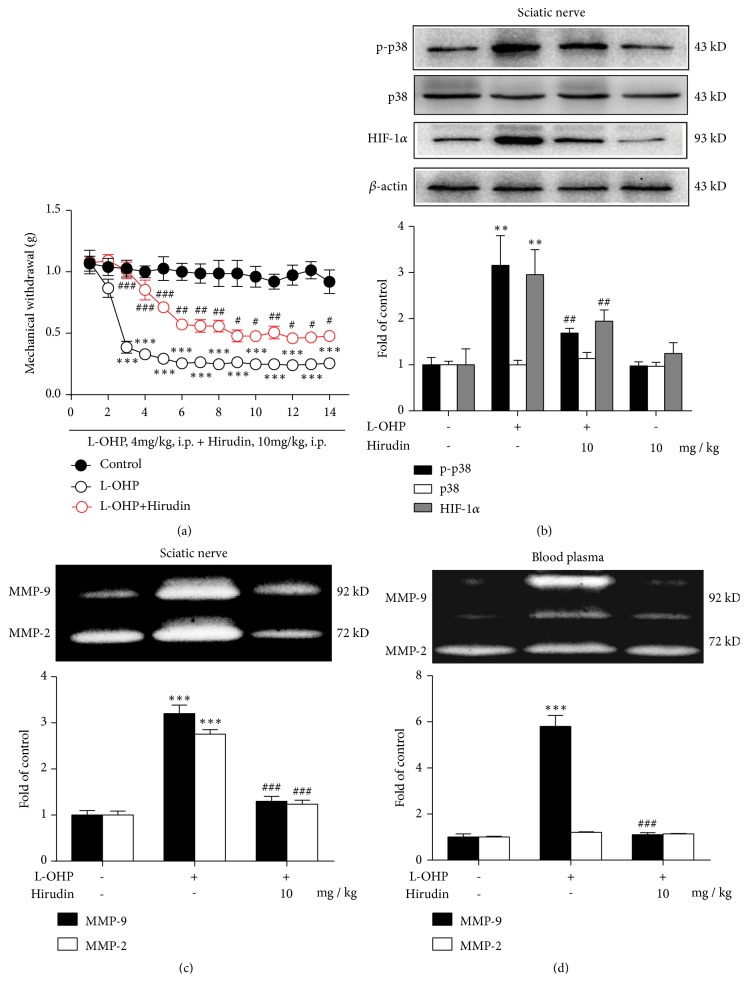
Hirudin can significantly suppress the overexpression of p-p38, HIF-1*α* and MMP-9/2 induced by L-OHP and attenuate CIPN. (a) Mechanical thresholds after four times injection of vehicle or L-OHP (4 mg/kg,* i.p.*, injected on 1, 4, 8 and 11 day) (n = 6). (b) The expression of p-p38, p38 and HIF-1*α* in sciatic nerve of mice on 14 day after four times injection of vehicle or L-OHP (4 mg/kg,* i.p.*, injected on 1, 4, 8 and 11 day) (n = 4). The activity of MMP-9/2 in sciatic nerve (c) and the blood (d) of mice on 14 day after four times injection of vehicle or L-OHP (4 mg/kg,* i.p.*, injected on 1, 4, 8 and 11 day) (n = 4). Hirudin (10 mg/kg,* i.p.*) was administrated once per day till 14 days. The first administration of hirudin was from the day before the CIPN model was built. Significant difference was revealed following one-way ANOVA (∗*P* < 0.05, ∗∗*P *< 0.01, ∗∗∗*P *< 0.001* vs*. control; ^#^*P *< 0.05, ^##^*P *< 0.01, ^###^*P *< 0.001* vs*. L-OHP group; Bonferroni post hoc tests).

Previous results have shown that L-OHP could induce the expression of HIF-1a and activation of MMP-9/2, so we screened compounds that may interfere with the above targets at the same time. The convincing article demonstrates that chemotherapy can induce thrombosis, and thrombosis can activate p38, which is very important for TF expression. So we thought of using a classical anticoagulant Hirudin to inhibit thrombosis, thereby inhibiting HSP70 release and TF expression. We evaluated the effect of Hirudin on L-OHP induced CIPN behavior in mice. As shown in Figure 4, Hirudin could significantly inhibit the mechanical hypersensitivity (Figure 4(a)) and significantly inhibit the expression of p-p38, HIF-1*α* and activity of MMP-9/2 in sciatic nerve and blood, but did not affect the expression of p38 (Figures 4(b)–4(d)).

### 3.5. p38 Inhibitor SB203580 Decreased the Expression of TF in RAW 264.7 Cells by L-OHP Treated

**Figure 5:**
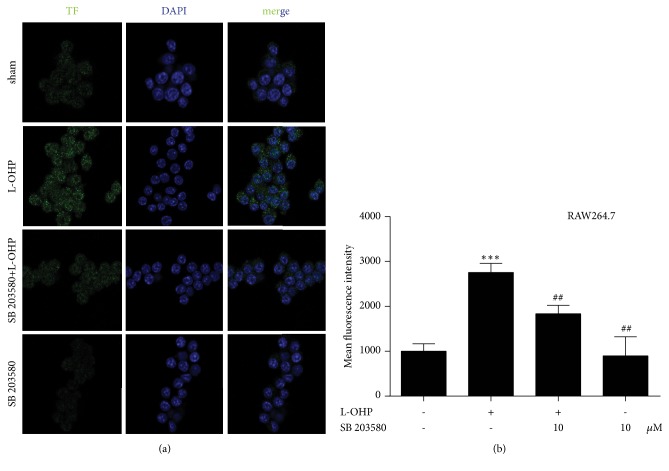
p38 inhibitor SB 203580 decreased the expression of TF in RAW 264.7 cells L-OHP treated. (a)(b) The expression of TF in RAW 264.7 cells after administrated with vehicle or L-OHP (10 *μ*M) or p38 inhibitor SB 203580 (10 *μ*M) (n = 4). Significant difference was revealed following one-way or two-way ANOVA ∗*P* < 0.05, ∗∗*P* < 0.01, ∗∗∗*P *< 0.001* vs*. control; ^#^*P *< 0.05, ^##^*P *< 0.01, ^###^*P *< 0.001* vs*. L-OHP group; Bonferroni post hoc tests).

Considering that phosphorylation of p38 plays an important role in the expression of HIF-1*α*, we measured whether p38 participated in the expression of TF induced by L-OHP. As shown in Figure 5, L-OHP significantly increased the phosphorylation of p38 in RAW 264.7 cells, which was abolished by the p38 inhibitor SB 203580.

## 4. Discussion

TF, especially which was expressed on monocytes and macrophages in the blood, has been reported to be significantly elevated by chemotherapy [25]. Previous studies have focused on the role of TF in hypercoagulable diseases [26], since TF is the initiator of exogenous coagulation [27]. Normally, TF is expressed at low levels in both blood cells and vascular endothelium. After chemotherapy, TF increases rapidly on the surface of monocytes and macrophages, inducing small thrombosis, leading to the high risk of thrombotic diseases, such as pulmonary embolism [28]. However, whether TF and its triggered downstream signals are involved in CIPN has not been reported. Our study found that a single L-OHP treatment could induce rapid and intense TF overexpression in the blood and sciatic nerve within a few hours. Moreover, the high expression of TF can be maintained for a long time, even after drug withdrawal (Figures 1(c)–1(f)).

TF, as a 47,000-molecular-weight protein, can be induced by chemotherapy in the sciatic nerve within a few hours, suggesting that this high expression may be due to the carrying and aggregation of blood cells in the sciatic nerve microenvironment. Previous studies have found that chemotherapy triggers chemokines overexpression in the peripheral nervous system and macrophage infiltration [29, 30]. Therefore, we examined the effect of L-OHP on the expression of TF on macrophages. The results showed that L-OHP treated macrophage RAW264.7 could also express TF rapidly, and the increased expression time was consistent with the blood expression trend of the whole animal laboratory. Our immunofluorescence data also confirmed the over-expression of TF on macrophages (Figures 5(a) and 5(b)). We then investigated which signaling pathways might mediate the high expression of TF on macrophages induced by chemotherapy. A large number of articles show that activation of TLR-4 -p38 signal is an important upstream signal of TF expression [31].

In sepsis models, LPS treatment can rapidly induce overexpression of TF in monocytes, macrophages, and endothelial cells [31]. At the same time, convincing studies have shown that TLR-4 plays a pivotal role in CIPN [32]. Inhibiting or knocking out TLR-4 receptors can significantly alleviate CIPN [33]. As a downstream molecule of TLR-4 receptor, p38 is a member of MAPK family and plays an important role in the expression of inflammatory and TF induced by TLR-4 [34, 35]. Our results showed that p38 in blood cells and sciatic nerve was activated rapidly after chemotherapy, and WB results of macrophages in vitro confirmed this. Next we examine what factors may trigger TLR-4-p38 activation. A recent study found that chemotherapy-induced gastrointestinal bacteria break through the intestinal immune barrier and enter the circulatory system, especially LPS release into the circulatory system and peripheral nervous system may be an important cause of chemotherapy pain [36]. It is well known that LPS can induce the expression of TF on macrophages, so the high expression of TF on macrophages may be due to LPS-induced is a reasonable speculation [7]. But based on the fact that L-OHP can induce high TF expression in a few hours, and chemotherapy-induced immunosuppression and gut barrier destruction may take days, we continue to examine other possible mechanisms. Chemotherapy is a very stressful stimulus for cells. Previous studies have shown that HSP70 can be rapidly released into the extracellular space under stress and acts as an endogenous ligand to activate TLR-4 receptors [37, 38]. Therefore, we examined the expression of HSP70 in serum before and after chemotherapy and found that the level of HSP70 increased significantly within two hours after chemotherapy. Moreover, the increase of HSP70 in serum was earlier than the increase of TF in blood cells. The data on macrophages in vitro are also consistent with those in vivo; chemotherapy can rapidly induce macrophages to release HSP70 (Figure 2(b)).

We then examined the hypothesis that TF over-expression could induce ischemia-hypoxia-related molecular events. Our results showed that HIF-1a and its downstream MMP-9/2 activity in the sciatic nerve and macrophage were significantly increased after chemotherapy, and their time of increase was consistent with that of TF and p38. These data suggest that there may indeed be a TLR-4-p38- TF -HIF-1*α* axis activation in the sciatic nerve microenvironment during the pathological process of CIPN. Next, we will validate our findings using compounds targeting the molecules mentioned above and examine their effects on CIPN (Figures 3(a)–3(d))

As mentioned above, HSP70 release induced by cell stress and TF overexpression mediated by p38 activation are two important molecular events [37]. ROS accumulation in cells may be the central mechanism [39], because chemotherapy can induce mitochondrial DNA damage and ROS elevation [40]. ROS can induce HSP70 release and p38 activation [41]. Previous studies have indicated that Hirudin modulates thrombosis through the inhibition of coagulation [42, 43], so we use Hirudin to examine its effects on the molecules on this axis. The data showed that Hirudin could significantly inhibit the over expression of p-p38, HIF-1*α* and activity of MMP-9/2 induced by L-OHP in vivo (Figures 4(a)–4(d)). However, it should be noted that direct anticoagulation may help to alleviate chemotherapy pain, but considering that chemotherapy can lead to thrombocytopenia in many patients, coagulation dysfunction may exist in chemotherapy patients at the same time, and direct anticoagulation for the treatment of CIPN may cause cardiovascular accidents in patients with hypocoagulability. Further studies are needed on the experimental study of anticoagulant drugs in the treatment of CIPN.

## 5. Conclusion

CIPN is one of the common side effects of chemotherapy and severely affects patients' compliance with chemotherapy. Our study reveals that TF, coagulation-related factors expressed by macrophages in the blood, may be induced by chemotherapy, and CIPN can be induced by macrophage recruitment and activated in the affected sciatic nerve. By stimulating the hypoxia-related signaling pathway, the increase of MMP-9 activation can be rapidly induced, and the high expression of TF in macrophages may be mediated by HSP70-TLR-4-p38 signaling pathway. We also tried to validate a new therapeutic strategy by supplementing hirudin to improve the hypoxic state of peripheral nerve microenvironment, thereby inhibiting the high expression of HIF-1alpha and MMP-9 in sciatic nerve to relieve pain. In view of the excellent clinical safety and low price of hirudin, our results may provide a new idea for the treatment of CIPN.

## Data Availability

The data used to support the findings of this study are available from the corresponding author upon request.
